# Astrocyte autophagy-pyroptosis crosstalk in Alzheimer’s: insights from knowledge graphs

**DOI:** 10.1186/s12883-026-04793-w

**Published:** 2026-03-28

**Authors:** Ting Liu, Zhisheng Huang, Hongyun Qin

**Affiliations:** 1 Department of Central Laboratory, Pudong Gongli Hospital, Shanghai University of Medicine & Health Sciences, No. 219 Miaopu Road, Shanghai, 200135 China; 2 Clinical Research Center for Mental Disorders, Shanghai Pudong New Area Mental Health Center, Tongji University School of Medicine, No. 165 Sanlin Road, Shanghai, 200124 China

**Keywords:** Alzheimer’s disease, Knowledge graph, Astrocytes, Autophagy, Pyroptosis

## Abstract

Astrocytes play a crucial role in brain health and are implicated in various aspects of Alzheimer’s disease (AD) progression. However, the mechanism underlying astrocyte autophagy and pyroptosis in AD remains unclear. To advance this knowledge, we constructed a knowledge graph of AD and dementia (AdDKG) by integrating literature-derived data with the Systematized Nomenclature of Medicine – Clinical Terms (SNOMED CT). The core knowledge base, *addkg*, was created through named entity recognition and semantic annotation of 172 283 articles using a concept identifier (CI-er) tool. AdDKG knowledge graph extends *addkg* knowledge base by incorporating SNOMED CT to standardize entities and enrich their relations and attributes. To validate the usefulness of AdDKG, we designed a case study to investigate the roles of astrocyte autophagy and pyroptosis in AD. The query results demonstrated that AdDKG precisely retrieves and prioritizes highly relevant articles, enabling focused investigation of mechanistic relations that would otherwise require extensive manual curation. Further detailed analysis revealed that abnormal autophagy, pyroptosis-induced inflammation, and their interplay are pivotal in AD progression. These findings highlight the utility and efficiency of AdDKG as a research tool for AD research, through which we elucidated the autophagy-pyroptosis interaction in astrocytes as a critical AD mechanism.

**Supplementary Information:**

The online version contains supplementary material available at 10.1186/s12883-026-04793-w.

## Introduction

Dementia is a general term for a decline in mental ability severe enough to interfere with daily life. Among the spectrum of dementias, Alzheimer’s Disease (AD) stands out as the prevalent form. AD is responsible for an estimated 60 to 80 percent of dementia cases, particularly affecting the elderly population [1]. As there is currently no cure for AD, it poses substantial challenges for affected families and societies [2]. Therefore, understanding the mechanisms of AD is crucial for developing effective therapeutic strategies.

Current research indicates that the pathological changes in AD begin with the deposition of amyloid-beta (A$\beta$), followed by Tau phosphorylation (p-Tau) and brain atrophy [3]. However, all interventions aimed at reducing A$\beta$ deposition have failed in clinical applications [4]. Consequently, there has been a continuous investigation into the toxic effects of A$\beta$ on neurons and their surrounding environment [5].

Autophagy is a key cellular process that maintains homeostasis by sequestering and degrading dysfunctional cytoplasmic components. It has been reported that autophagic processes are involved in the degradation of internalized A$\beta$ [6], a function previously studies mainly in neurons and microglia cells [6–8].

Importantly, growing evidence highlights the critical role of astrocytes, the primary homeostatic cells in the brain, in AD pathogenesis. While essential for neuronal support, astrocytes in AD exhibit dysfunctional autophagy, which is associated with the accumulation of A$\beta$ [9] and the spread of Tau pathology [10]. Concurrently, these astrocytes often adopt a proinflammatory phenotype [11]. Our previous experiments proved that pyroptosis of astrocytes promoted inflammatory responses and A$\beta$ was released upon activation by high-level or prolonged A$\beta$ treatment [12]. However, the mechanistic relationship between neuroinflammation and astrocyte autophagy in AD remains unclear.

To advance this knowledge, this study aims to construct a knowledge graph that integrates scientific articles focusing on AD and dementia. It will serve as a systematic platform for knowledge retrieval and inference, thereby enabling a deeper exploration of the interplay between neuroinflammation and astrocyte autophagy in the pathogenesis of AD.

Knowledge graphs are a powerful analytical tool that integrates information as a network of interconnected nodes and edges [13]. Nodes represent entities, while edges denote the relationships between them. This structured approach not only organizes diverse data into a flexible and adaptable framework but also leverages network analysis methods to uncover underlying patterns and insights that may remain obscured in traditional data representations [14].

Knowledge graphs have been widely adopted and have shown promising applications in the medical domain, such as drug discovery, disease classification, and combination therapies [15]. Considering the advantages of knowledge graphs in integrating disparate resources and performing semantic searches, we applied this technique to construct an Alzheimer’s disease and Dementia Knowledge Graph (AdDKG) by semantically integrating literature-derived data with the Systematized Nomenclature of Medicine – Clinical Terms (SNOMED CT).

In this study, we used XMedlan, an advanced Xerox Concept Identifier (CI-er) tool, to perform named entity recognition and semantic annotation of the titles and abstracts of publications retrieved from the PubMed database [16]. Named entities were recognized by CI-er by matching words to the associated entity labels in the SNOMED CT thesaurus and further tagging them with a unique entity Uniform Resource Identifier (URI). Detailed annotations and relations between recognized entities and source articles are represented in a triplet format: *subject-predicate-object*. These literature-derived data were stored in the *addkg* knowledge base, which was then integrated with SNOMED CT through entity alignment to standardize clinical terms and enrich semantic relations. This process enhanced both contextual attributes and relational depth, ultimately forming the more comprehensive AdDKG knowledge graph.

We performed a semantic query to retrieve meaningful results, thereby substantiating the usefulness of AdDKG. The query results confirmed that AdDKG precisely retrieves and prioritizes highly relevant articles, enabling focused investigation of mechanistic relations that would otherwise require extensive manual curation. A deeper analysis highlighted the critical role of abnormal autophagy, pyroptosis-induced inflammation, and their dynamic interplay in AD progression. Therefore, we believe that the resources and insights from this work will greatly benefit biomedical researchers in AD by providing a relatively comprehensive and interconnected dataset to advance their studies.

## Methods

**Fig. 1:**
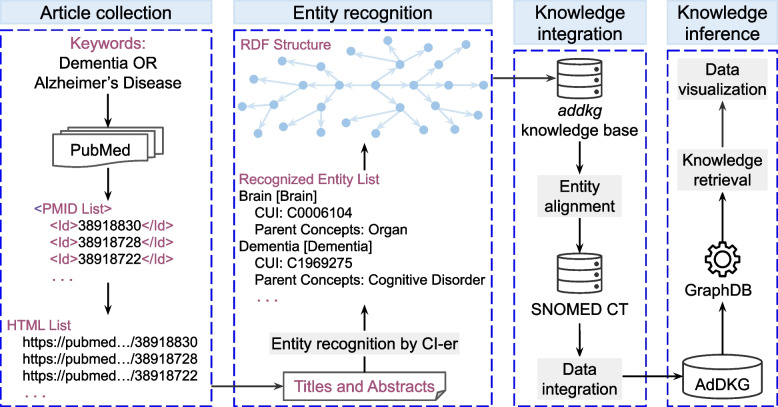
The workflow for constructing the AdDKG knowledge graph. The pipeline comprises four stages. (1) Article collection. Relevant articles were retrieved from PubMed, generating PMID and URL lists. (2) Entity recognition. Biomedical concepts were extracted and structured into RDF triples using the CI-er tool. (3) Knowledge integration. Extracted entities were semantically aligned with the SNOMED CT ontology. (4) Knowledge inference. The completed AdDKG was hosted on GraphDB, enabling querying, visualization, and knowledge discovery

This section outlines the workflow for constructing the AdDKG knowledge graph, as illustrated in Fig. 1, which consists of four steps: article collection, entity recognition, knowledge integration, and knowledge inference.

### Article collection

We initiated our study by conducting a comprehensive search of PubMed for peer-reviewed articles published between January 1, 2009, and December 31, 2023. Using the Entrez Programming Utilities ESearch API [17], we executed queries for two keywords: Alzheimer’s disease and dementia. This search strategy yielded 172 283 unique PubMed IDs (PMIDs) of relevant publications. PMIDs serve as persistent, unique identifiers that facilitate reliable data tracking throughout the analysis pipeline. Given that, titles and abstracts provide concentrated conceptual information relevant to our research objectives, while full-text articles often contain extensive methodological details that are beyond our current scope. For subsequent analysis, we therefore focused on the titles and abstracts of these publications rather than the full-text content. Specifically, we extracted the titles, abstracts, and other metadata components pairs with the 172 283 PMIDs for further entity recognition and semantic annotation.

### Entity recognition

Entity recognition processes titles and abstracts to recognize and classify named entities. Since sentences in titles and abstracts often feature multiple heterogeneous entities, an integrated pipeline is required to handle this complexity. To address this, we used the CI-er tool [16] for named entity recognition and semantic annotation of biomedical text. The tool was previously evaluated on gold-standard biomedical datasets, achieving competitive performance with precision, recall, and F1-score all around 92% [16]. Its pre-validated nature provides a high-quality baseline for our entity recognition, ensuring consistency and accuracy without the need for comprehensive re-validation.

**Fig. 2:**
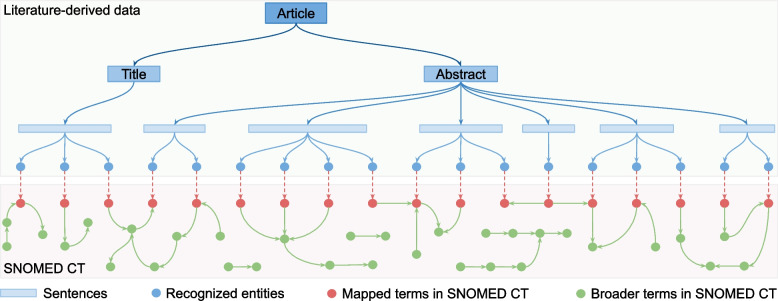
Schema of knowledge representation and entity mapping within AdDKG. The diagram illustrates the data model for structuring literature-derived knowledge. An article (Title and Abstract) is decomposed into individual sentences, represented as blue segments. Within sentences, recognized entities are denoted by blue dots. These entities are mapped to corresponding standardized terms (red dots) in SNOMED CT, which contains a broader network of biomedical terms and relations (green dots and arrows)

The CI-er performs term identification through sequential strategies combining typographical, morphological, orthographical, and misspelling normalization. These filtering operations implemented primarily via compositions of finite-state transducers [13, 16], ensuring robust entity recognition prior to knowledge graph construction. Furethermore, it features a terminology compiler that integrates the SNOMED CT thesaurus (2022 Edition) as its term base to identify and classify entities. Using CI-er, entities in the token stream are recognized and mapped to SNOMED CT. As a result, 148 864 unique entities were identified through this process. The relations among articles, sentences, entities, and terms in SNOMED CT are illustrated in Fig. 2. These relations are structured in RDF format and then stored in the *addkg* knowledge base for subsequent knowledge integration and graph analysis.

### Knowledge integration

Knowledge integration is crucial as it involves synthesizing information from diverse sources to gain a comprehensive database. Contemporary efforts in knowledge integration rely on third-party databases such as the SNOMED CT thesaurus, which offer standardized terminologies and ontologies to unify medical concepts across domains [18]. Entity alignment is an essential process of knowledge integration that aims to unify and connect different entities or terms from diverse databases that refer to the same one. To interconnect and integrate various datasets, we aligned entities in the *addkg* knowledge base with the corresponding terms in the SNOMED CT database by matching their labels and URIs. In this way, integrating the two databases with different sources and coverage enhances the depth and specificity of our knowledge graph. While a significant number of SNOMED CT terms are not directly aligned, as illustrated in Fig. 2, they still contribute valuable term relations and semantic attributes, enriching the overall knowledge structure.

### Knowledge inference

The AdDKG knowledge graph is stored in the graph management and analysis platform GraphDB [19], which enables users to perform SPARQL queries to retrieve and infer knowledge, as well as to visualize data (see Fig. 1). SPARQL is a powerful language used for querying and manipulating RDF data stored in the graph database [20]. It enables users to execute complex queries, uncovering hidden relationships, patterns, and insights from the integrated knowledge graph.

To demonstrate this knowledge inference capability, we present a case study in the Results section to investigate the crosstalk between autophagy and pyroptosis within astrocytes in AD. This example showcases how structured queries can extract meaningful biological relations from the literature-derived knowledge base.

To facilitate semantic retrieval and enhance reproducibility, we provide a collection of ready-to-use SPARQL query templates in the Supplementary File. These templates, which cover tasks from basic entity retrieval to complex relational inference, are annotated to guide users in customizing them for their own research needs.

## Results

### Statistics of AdDKG

We constructed the AdDKG knowledge graph in the GraphDB database by integrating two distinct datasets derived from two types of resources: (*i*) the *addkg* knowledge base, which is a semantically annotated collection of scientific publications by CI-er, offering up-to-date research on AD and dementia; and (*ii*) the SNOMED CT thesaurus, which is a widely recognized and publicly accessible database, providing well-structured information on the underlying biomedical terms and relations.

The resulting AdDKG knowledge graph, as detailed in Table 1, consists of 261 million triples, with 251 million originating from the *addkg* knowledge base and ten million derived from the SNOMED CT thesaurus. The *addkg* dataset comprises a total of 148 864 named biomedical entities identified from 172 283 scientific publications, whereas the SNOMED CT thesaurus contains 491 601 biomedical terms. Notably, the concept of ‘dementia’ stands out as the most frequently annotated term, appearing 228 552 times throughout the annotations.

**Figure:**
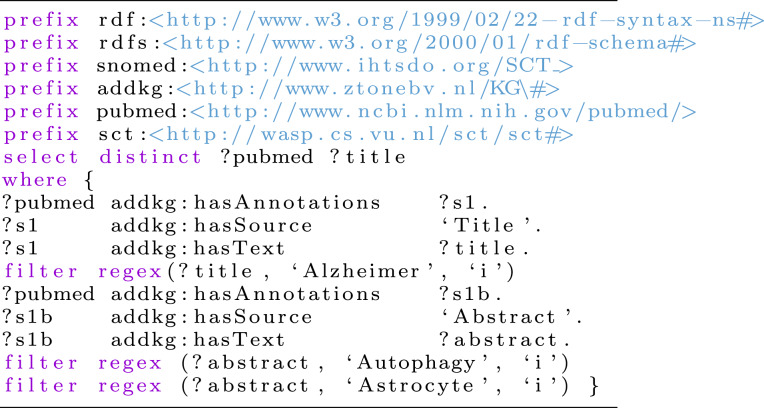
**Listing 1** Query protocol to retrieve articles on the role of astrocytes in the autophagic process in AD

**Table 1:** The term and triple statistics of AdDKG

Dataset	Papers	Terms	Triples	
*snomed ct*	N/A	491 601	10 375 127	
*addkg*	172 283	148 864	250 920 458	
Total	172 283	640 465	261 295 585	
Most frequently annotated concept in *addkg***:**				
			Occurrences	228 552
			ID	192180006
			Label	Dementia: [unspecif] or [presenile NOS ( &including presenile psychosis NOS)] or [primary degenerative NOS] or [senile NOS (including senile psychosis NOS)] or [senile depressed or paranoid type] (disorder)

### Validation of AdDKG

In this section, we presented a case study of AdDKG to demonstrate the usefulness of the AdDKG knowledge graph in AD research. Through semantic literature mining and analysis, we conducted a comprehensive investigation into the interaction between autophagy dysfunction and astrocyte pyroptosis in AD.

#### Semantic literature mining

Autophagy, a cellular self-degradation process essential for maintaining homeostasis, stress adaptation, and survival, has been implicated in AD pathogenesis. Notably, neuronal autophagy dysfunction contributes to synaptic impairment in AD [21]. Given this connection, we sought to investigate the correlation between autophagic dysregulation and AD progression. To achieve this, we first designed a SPARQL query (see the code presented in Listing 1) to identify publications linking autophagic deficits to AD and dementia, retrieving 694 relevant articles (see SI Table 1).

It is well known that neurons lack intrinsic energy storage capacity and critically rely on metabolic support from their surrounding microenvironment, particularly astrocytes [22]. These glial cells play a pivotal role in neuronal development, metabolism, and synaptic transmission and plasticity [23], forming essential components of brain homeostasis. Our previous work [12] demonstrated that A$\beta$ induces astrocytes’ pyroptosis in vitro, concomitantly increasing the release of inflammatory cytokines (IL-1$\beta$ and IL-18) and the aggregation of pathogenic proteins (A$\beta$ and p-Tau). These findings highlight the critical involvement of astrocyte-mediated inflammation in AD pathology, which is of particular interest. Building upon these findings, we investigated the regulatory role of astrocytes’ autophagy in AD pathogenesis.

To further investigate the crosstalk between autophagy and pyroptosis in astrocytes during AD, we refined the previously established SPARQL query framework (see Listing 1) by adding ‘pyroptosis’ and ‘astrocyte’ as new key terms. This semantic expansion returned 12 high-relevance publications from the previously identified set of 694 articles linking autophagy to AD. The query was based on the co-occurrence of these key terms within the retrieved text, ensuring direct relevance to our research focus. It thus provided a highly focused corpus for our subsequent in-depth analysis of the autophagy-pyroptosis crosstalk within astrocytes in the context of AD. This curated collection included 4 review articles and 8 original research articles, with a language distribution of 11 English and 1 Chinese. The complete details of these 12 articles are provided in SI Table 2.

#### The interplay between astrocyte autophagy and pyroptosis in AD

**Fig. 3:**
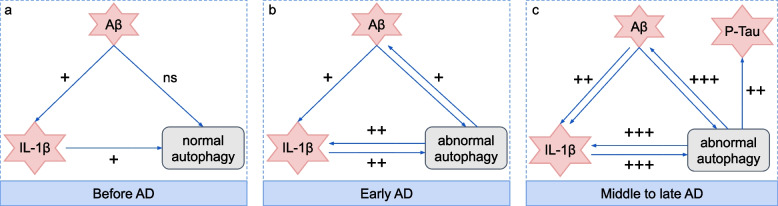
A stage-dependent model of the interplay between autophagy and pyroptosis within astrocytes in AD. Before AD, IL-1$\beta$ from pyroptosis activates normal autophagy without affecting A$\beta$ deposition. In early AD, sustained IL-1$\beta$ increase leads to abnormal autophagy, failing to clear A$\beta$ and promoting pyroptosis and A$\beta$ deposition. In the middle to late AD, pyroptosis and A$\beta$ deposition intensify, along with p-Tau accumulation. Disease phases and their key features were identified from retrieved results

We conducted an in-depth analysis of the 12 retrieved articles, detailing their research topics and proposed mechanisms in Table 2. Through expert synthesis of the evidence contained in these publications, we constructed a conceptual mapping of the underlying biology. This mapping reveals a dynamic, stage-dependent interplay between astrocytes’ autophagy and pyroptosis in AD pathogenesis, as visually summarized in Fig. 3.

**Table 2:** The 12 retrieved articles concerning the interplay between autophagy and pyroptosis within astrocytes in AD

PMID (Ref.)	Topic	Possible mechanisms
26452999 [23]	A$\beta$causes dendritic spine loss	A$\beta$reduces astrocytic TSP-1 secretion via autophagy activation, leading to dendritic spine loss
24330807 [24]	Inflammation triggers autophagy	IL-1$\beta$co-localized with p62 and LC3 in the tri-cultures of neurons, astrocytes, and microglia, demonstrating that pro-inflammatory cytokines induce autophagy
25596147 [25]	Wogonin boosts A$\beta$clearance and inhibits p-Tau	Wogonin inhibits the phosphorylation of mTOR and subsequently induces autophagy, thereby promoting A$\beta$clearance and p-Tau accumulation of primary cortical astrocytes
26235241 [26]	A$\beta$deposition decreases autophagy	In AD mice, GFAP/LC3+ co-localization declined from 50% (15mo) to 20% (20mo), showing that A$\beta$deposition progressively impairs astrocytic autophagy
32985163 [27]	Presenilin-1 mutations in AD	The L286V mutation in the presenilin-1 gene induces impaired mitophagy in neurons
34433343 [28]	Neurotoxicants in AD pathogenesis	Neurotoxicants disrupt autophagic flux in neurons and glia through mTOR signalling activation
34139302 [29]	EGFR inhibitors attenuate AD	EGFR inhibitors attenuate astrocyte reactivation by inhibiting mTOR and promoting autophagy
35008592 [30]	Neurodegeneration and astrogliosis	Astrogliosis correlates with A$\beta$deposition and excessive p-Tau in hippocampus
35008671 [31]	HSV-1 disrupts neuron-glial oneness	Astrocytes exhibit multifaceted responses to HSV-1 infection, involving inflammatory activation, oxidative stress, A$\beta$deposition, p-Tau accumulation, apoptosis, and autophagy
36729287 [32]	Impaired autophagy in immortalized astrocytes	Autophagy impairment involves proteasome dysfunction, disrupted autophagy proteins, mitochondrial defects, and neuroinflammation
36755969 [33]	AVE 0991 inhibits astrocyte pyroptosis	AVE 0991 promotes autophagy by increasing LC3II/I ratio and Beclin-1 expression while decreasing p62 level, thus inhibiting astrocytes pyroptosis
35780157 [34]	Spermidine reduces A$\beta$deposition	Spermidine enhances astrocyte autophagy, clearing A$\beta$and lowering IL-1$\beta$

##### Phase 1: Pre-AD stage

Before AD onset, IL-1$\beta$ – an inflammatory protein released through astrocyte pyroptosis and activated by microglia – may initiate autophagy [12]. This autophagic response not only reduces the deposition of A$\beta$ [31] but also inhibits the maturation and secretion of the proinflammatory cytokines IL-1$\beta$ and IL-18 [24]. A delicate balance between these two cellular processes is crucial to prevent the pathological A$\beta$ aggregation.

##### Phase 2: Early AD stage

Inhibition of mTOR using rapamycin [24], natural product wogonin [25], or exogenous low doses of A$\beta$(20$\mu$M) [24] has been reported to activate autophagy. This enhanced autophagic activity is believed to promote A$\beta$ clearance, consequently reducing p-Tau of primary cortical astrocytes [25], a key step in the formation of neurofibrillary tangles in AD. Concurrently, A$\beta$ deposition triggers a mild increase in IL-1$\beta$ without the accumulation of acidic vesicles [24]. These findings suggest that A$\beta$ primarily induces cellular pyroptosis rather than exerting an immediate effect on autophagy. While A$\beta$ deposition moderately enhances autophagic activity, the emergence of inflammatory responses indicates cellular decompensation, wherein autophagy fails to clear pro-inflammatory cytokines.

##### Phase 3: Middle to late AD

In the progression of AD, sustained IL-1$\beta$ elevation coincides with autophagy markers (p62 and LC3) accumulation in acidic vesicles, indicating that pyroptosis subsequently activates the autophagy [24]. This is further supported by in vitro evidence showing that prolonged exposure triggers astrocytic autophagy, resulting in reduced thrombospondin-1 (TSP-1) secretion and hippocampal neuron spine loss – effects reversible by the autophagy inhibitor 3-methyladenine [23].

Notably, p62 accumulation in astrocytes of AD mouse models marks a critical transition in A$\beta$-induced astrocyte activation, shifting from neuroprotective autophagy initiation to pathogenic autophagic flux impairment [26, 30, 32]. This pathological shift creates a paradoxical feedforward loop: although A$\beta$ initially stimulates astrocytes’ autophagy, the subsequent flux failure prevents A$\beta$ clearance, ultimately driving the extracellular deposition of A$\beta$ [24, 27]. Moreover, sustained autophagy in astrocytes triggers pyroptosis, leading to the release of neuroinflammatory factors.

As AD progresses, a mutually reinforcing vicious cycle occurs between dysfunctional autophagy and pyroptosis. Autophagic dysfunction fails to clear A$\beta$, leading to its deposition. The accumulating A$\beta$ then triggers astrocyte pyroptosis, resulting in the release of pro-inflammatory cytokines (e.g., IL-1$\beta$). Conversely, sustained inflammation (evidenced by LC3/IL-1$\beta$ co-localization) further worsens the impaired autophagy, which in turn aggravates cellular impairment and ultimately induces pyroptosis in astrocytes. This vicious cycle, where inflammation and autophagy dysfunction mutually reinforce each other, not only exacerbates A$\beta$ deposition but also accelerates p-Tau accumulation, thereby driving the progression of AD pathology. As pathological astrocytes accumulate during disease progression, their production of protective substances (e.g., TSP-1) is markedly reduced, consequently compromising neuronal structural integrity and inducing neuron death.

##### Molecular regulation of autophagy

Dysregulated autophagy is a central pathogenic mechanism in AD. This impaired autophagic flux initiates a vicious cycle with pyroptosis and drives subsequent neurodegenerative cascades. A deeper understanding of the molecular regulation of autophagic flux is therefore critical. Here, we synthesize the key genetic, environmental, and pharmacological factors that disrupt autophagy in the progression of AD.

On the genetic front, loss of presenilin-1 (PS1) gene function (a catalytic unit of the $\gamma$ secretase) triggers a pathogenic cascade characterized by A$\beta$ deposition, autophagic dysfunction, neuronal degradation, and chronic neuroinflammation [27]. Beyond genetic factors, environmental factors significantly contribute to autophagy dysregulation, with neurotoxic metals (e.g., arsenic, cadmium, copper, iron, manganese, and methylmercury) disrupting critical autophagic markers including mTOR, Beclin-1, and LC3 [28]. These metal-induced alterations create a permissive environment for AD pathogenesis.

**Fig. 4:**
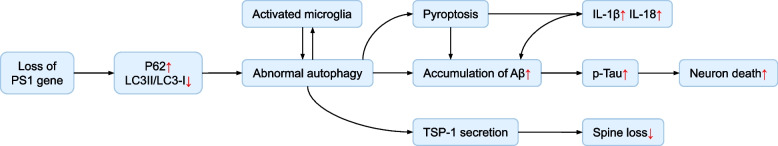
Mechanisms of autophagy-pyroptosis crosstalk in AD astrocytes involving genetic risk factors. These genes dysregulate autophagy, leading to A$\beta$ deposition, pyroptotic cell death, and reduced TSP-1 secretion, while also contributing to microglial activation. Then microcytes activate astrocytes to abnormal autophagy, increase the excessive accumulation of A$\beta$, induce a large amount of p-Tau, and reduce pyroptosis to release large amounts of inflammatory marker IL-18 and IL-1$\beta$, eventually leading to the death of neurons. Core components and interconnected causal relations were identified within sentences retrieved from the AdDKG

In this context, emerging evidence also highlights the critical role of pharmacological agents in modulating autophagy. Notably, EGFR inhibitors stimulate autophagy by inhibiting mTOR activity, which in turn removes A$\beta$ and alleviates neuroinflammation [29]. This stimulation mitigates cognitive decline in APP/PS1 transgenic AD mice [29]. Similarly, AVE 0991, an agonist of the Ang-(1-7) receptor, restores astrocyte autophagy by upregulating Beclin-1/LC3 expression while downregulating p62 levels [33]. This restoration suppresses A$\beta$-induced neuroinflammation by inhibiting the release of NLR family pyrin domain containing 3 (NLRP3) inflammasome, a key factor of astrocyte pyroptosis [12]. Most importantly, the autophagy activator spermidine has been found to reduce microglia-mediated neuroinflammation in APP/PS1 mice by activating autophagy pathways [34]. This coordinated mechanism, synthesized from the retrieved evidence, is illustrated in Fig. 4. Together, these findings indicate the potential molecular mechanisms of autophagy in AD pathogenesis.

## Discussion

### Advantages of AdDKG

The primary strength of AdDKG lies in its capacity to semantically integrate vast amounts of dispersed scientific literature, enabling researchers to efficiently uncover novel insights. In this study, we constructed AdDKG to explore the interactions between pyroptosis and autophagy in astrocytes, thereby deepening our understanding of AD pathogenesis. Traditional manual review of 172 283 articles on AD and dementia would be prohibitively time-consuming, but AdDKG automates this process by systematically annotating and connecting key entities. It not only streamlines and accelerates the integration of large-scale literature but also allows users to retrieve highly relevant publications by tracking entity co-occurrences, significantly reducing the time researchers would otherwise spend manually reviewing extensive datasets.

Another key advantage of AdDKG is its logical framework, which prioritizes the strongest associations while filtering out weaker or less relevant connections. This selective approach minimizes information overload, enabling researchers to focus on high-impact findings. As presented in Validation of AdDKG section, our analysis narrowed down a vast corpus of AD-related publications to a concise set of 12 highly pertinent articles discussing pyroptosis and autophagy in astrocytes. This demonstrates AdDKG’s ability to substantially improve retrieval precision without sacrificing coverage, ensuring that researchers spend less time sifting through irrelevant studies and more time on meaningful analysis. Additionally, this process not only facilitated the summarization of astrocyte-related mechanisms in AD but also highlighted gaps in current research, such as the limited number of studies on astrocyte functions, guiding future investigations.

The AdDKG knowledge graph supports users to perform a variety of queries, including: (*i*) filtering highly relevant domain articles; (*ii*) identifying co-occurring concepts across publications; (*iii*) retrieving sentences that target concepts co-occurred; (*iv*) discovering entity alignment information across databases; and (*v*) exploring core entities, their relations, and attributes. Through these queries, users can relate a concept in a specific study to other concepts within it, as well as to those in similar studies.

These features allow users to contextualize concepts within individual studies and across related research, fostering a more comprehensive understanding of AD mechanisms. By optimizing literature retrieval and knowledge synthesis, AdDKG serves as a powerful tool for accelerating discoveries in neurodegenerative disease research.

Beyond its functional advantages, the AdDKG knowledge base was designed to be findable, accessible, interoperable, and reusable. These core attributes align with the FAIR Guiding Principles [35], a community standard for high-quality scientific data management. By employing standardized identifiers and community ontologies, AdDKG enables precise discovery by both humans and machines, as well as seamless interoperability with other biomedical databases. To ensure transparency and facilitate data reuse, we provide multiple access pathways for data sharing. These features collectively ensure its quality and utility as a community-level knowledge resource.

### Validation of AdDKG

The validation of AdDKG was conducted through a comprehensive case study examining the roles of astrocyte autophagy and pyroptosis in AD pathogenesis. AdDKG successfully integrated 172 283 publications on AD and dementia, enhanced by semantic expansion using SNOMED CT thesaurus. This extensive knowledge base enabled efficient identification of the most relevant literature while maintaining broad coverage of the research domain.

To validate the usefulness and effectiveness of AdDKG, we designed a case study to investigate the role of astrocyte autophagy and pyroptosis in AD pathology (see Validation of AdDKG section). Several key findings emerged from this process, demonstrating the effectiveness of AdDKG. First, the AdDKG exhibited high precision in literature retrieval, successfully identifying 12 highly relevant articles from the extensive pool of 172 283 publications (see Table 2). Importantly, analysis of these core publications provided compelling evidence that impaired autophagy in astrocytes significantly contributes to AD pathogenesis, validating the AdDKG’s capability to extract biologically meaningful insights.

Furthermore, the structured knowledge graph enabled systematic investigation of the mechanistic interplay between astrocyte autophagy and pyroptosis through diverse dimensions: (*i*) the temporal dynamics of these processes during AD progression; (*ii*) the molecular cross-talk between autophagy and pyroptosis pathways; and (*iii*) potential therapeutic targets arising from their interaction. This multi-faceted analysis not only confirmed the AdDKG’s utility for exploring complex biological relations but also highlighted its value in generating research perspectives.

It is worth noting that, beyond the case study presented here, the quality of AdDKG could be further evaluated using frameworks like the FAIR principles [35] and the approaches proposed by Lan et al. [20] and Cortes et al. [36], which offer complementary dimensions for evaluating biomedical knowledge graphs.

### Many faces of astrocytes in AD

**Fig. 5:**
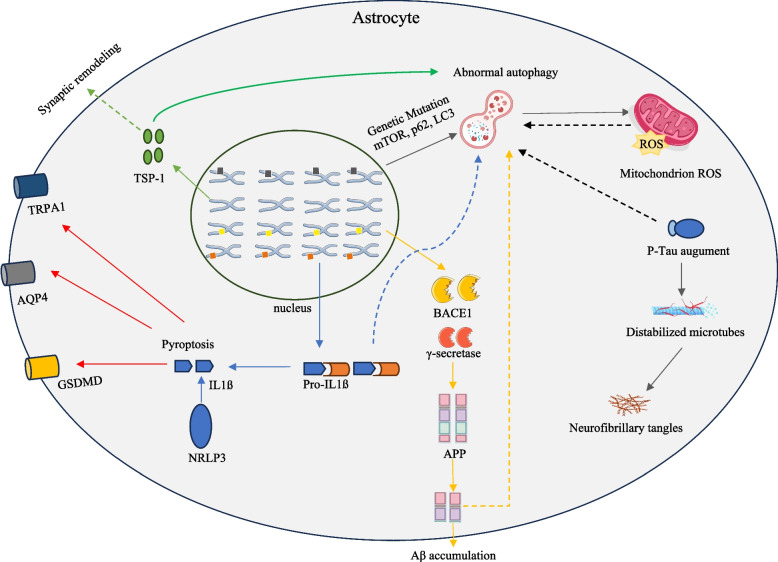
Schematic representation of the crosstalk between impaired autophagy and pyroptosis activation in AD astrocytes. This integrated overview is an expert-derived synthesis based on the stage-specific model (Fig. 3) and the mechanistic network (Fig. 4). Key pathways include: impaired autophagy leading to protein aggregation and mitochondrial ROS (black lines); pyroptosis activation and IL-1$\beta$-mediated inflammation (blue lines); A$\beta$ accumulation (yellow lines); and synaptic dysfunction via reduced TSP-1 (green lines). The AQP4/TRPA1/GSDMD dysfunction (red lines) represents complementary insights derived by experts. Solid lines represent signaling pathways associated with abnormal autophagy, and dashed lines represent those associated with normal autophagy

This study demonstrates that impaired autophagy in astrocytes critically contributes to AD pathogenesis, based on evidence synthesized from the analysis of 12 key publications (see Table 2). Building on this finding, we further dissect the mechanistic interplay between autophagy and pyroptosis of astrocytes in AD progression through three pivotal perspectives (see Fig. 5).

#### Abnormal autophagy drives AD

Autophagy dysregulation is a key driver of AD progression, affecting multiple cellular functions and involving complex mechanisms, including:

i. A$\beta$ deposition. Autophagy is a natural cellular process for A$\beta$ clearance; however, this degradation process becomes progressively impaired with increasing A$\beta$ concentrations or prolonged exposure (Fig. 3). Compound AVE 0991 repairs this impairment by reducing p62 levels, ultimately restoring autophagic flux and promoting A$\beta$ clearance [33].
ii. Tau dyshomeostasis. Beyond A$\beta$ deposition, aggregation of p-Tau into neurofibrillary tangles is a hallmark of AD pathology. Usually, autophagy clears misfolded A$\beta$ and p-Tau to maintain cellular proteostasis. Impaired autophagy causes p-Tau accumulation, especially in the middle to late stages of AD [25], aggravating neurodegeneration [37].
iii. Synaptic disruption. Impaired autophagy in astrocytes reduces the secretion of TSP-1 (an important substance in dendritic spines), lowering neuronal plasticity (see Fig. 4). This impairment also disrupts synaptic protein turnover, affecting its transmission, plasticity, and leading to neuronal atrophy. Restoring autophagy may protect synapses and decelerate cognitive decline [21, 23].
iv. Mitochondrial dysfunction. A$\beta$ deposition caused by impaired autophagy disrupts mitochondrial function and induces reactive oxygen stress (ROS) [31]. Oxidative stress damages cellular components and promotes neuronal cell death. While autophagy normally removes damaged components to mitigate ROS [28, 38], A$\beta$ deposition-impaired autophagy further compromises mitochondrial recovery.
v. Genetic regulation. Mutations in the APP, PSEN1, and PSEN2 genes – causative factors in early-onset AD – can indirectly impair autophagy [27]. In late-onset AD, the APOE-$\varepsilon$4 allele has also been implicated in modulating autophagic activity. These findings highlight the genetic-autophagy interplay in AD pathogenesis [27].

Taken together, autophagy dysfunction is a central driver of AD pathogenesis, linking A$\beta$ accumulation, p-Tau aggregation, synaptic damage, mitochondrial dysfunction, and genetic risk factors. Further investigation into autophagy modulation could unravel novel therapeutic interventions for AD.

#### Astrocyte pyroptosis in AD

During AD progression, activated astrocytes exacerbate neuroinflammation through pyroptosis, releasing pro-inflammatory cytokines such as IL-1$\beta$ and IL-18 (Figs. 3 and 4) [26, 39]. Evidence suggests that IL-1$\beta$ levels are chronically elevated in the AD brain, partly due to astrocyte pyroptosis [12]. The underlying mechanisms driving this process may involve:

i. Impaired autophagy fails to clear damaged mitochondria, resulting in NLRP3 inflammasome activation and subsequent pyroptosis [39, 40].
ii. Impaired astrocyte autophagy interacts with activated microglia, forming a pro-inflammatory feedback loop that further exacerbates pyroptosis [41, 42].
iii. Dysregulation of the water channel AQP4 and the ion channel TRPA1 on astrocyte membranes disrupts water and Ca$^{2+}$ homeostasis, leading to activation of protein phosphatase 2B and subsequent release of IL-1$\beta$. [43, 44].

In summary, neuroinflammation in AD involves a complex interplay of factors, including mitochondrial dysfunction, microglial activation, and membrane alteration caused by impaired autophagy, collectively contributing to AD progression through astrocyte pyroptosis and heightened inflammation [12]. As A$\beta$ accumulates, the rising number of pyroptotic astrocytes enhances IL-1$\beta$ release, further exacerbating abnormal autophagy and worsening AD pathology. Emerging immunotherapies such as Aducanumab - a monoclonal antibody targeting aggregated A$\beta$ - have shown clinically meaningful reduction in A$\beta$ plaques and slowing of cognitive decline in clinical trials [45]. These findings suggest inhibiting astrocytic pyroptosis could be a way to reduce neuroinflammation and potentially slow the progression of AD.

#### Autophagy-pyroptosis fuels AD

The dynamic interaction between autophagy and pyroptosis in astrocytes plays a pivotal role in AD pathogenesis. To uncover the underlying mechanisms driving AD progression, we delineated this interaction across different disease stages.

i. In the pre-AD phase, normal autophagy acts as a protective mechanism, clearing harmful protein aggregates (e.g., A$\beta$ and p-Tau) and recycling damaged organelles, thereby inhibiting pyroptosis [6]. Furthermore, by degrading proinflammatory mediators such as IL-1$\beta$, autophagy prevents excessive inflammatory responses that could otherwise trigger pyroptosis [24].
ii. As AD develops, chronic stress and cellular damage overwhelm autophagy, leading to pyroptosis activation [26]. Inflammatory cytokines (e.g., IL-1$\beta$) released during pyroptosis inhibit autophagy through two main mechanisms: direct suppression of autophagic activity or by caspase-mediated cleavage of autophagy-related proteins [32]. This forms a vicious cycle, where autophagy initially restrains inflammation but is subsequently impaired by sustained pyroptosis, leading to increased neurotoxicity.
iii. In advanced AD, impaired autophagy fails to clear damaged mitochondria, increasing oxidative stress and mitochondrial permeability transition. This dysfunction exacerbates pyroptosis, amplifying neuroinflammation and Tau pathology [46]. Additionally, pyroptosis-induced mitochondrial damage further suppresses autophagy while promoting p-Tau release [28] .

The interplay between autophagy and pyroptosis in AD involves a delicate balance between cellular protection and pathological feedback loops. Astrocytes, as key regulators of neuroinflammation, drive this process – their pyroptosis sustains chronic inflammation, while impaired autophagy accelerates neurodegeneration. Targeting this interaction, particularly by mitigating pyroptosis to restore autophagic function, may offer a promising therapeutic strategy for AD.

### Limitations and Future Work

The CI-er tool demonstrates competent performance in biomedical named entity recognition, enabling automated extraction of key entities (e.g., genes, proteins, drugs, and diseases) from unstructured text to construct knowledge graphs based on the co-occurrence of entities. However, its primary limitation lies in the inability to identify and characterize specific semantic relations between these entities (e.g., activates, inhibits, associated with, or biomarker for). While co-occurrence analysis can suggest potential associations, it fails to capture the nuanced biological interactions necessary for advanced applications such as drug mechanism interpretation, biomarker discovery, or predictive modeling. This restricts the utility of knowledge graphs, as relation-free networks lack the depth required for meaningful biomedical inference.

Similarly, the AdDKG framework currently relies predominantly on publication-derived data, which ensures scientific validity and facilitates reproducibility but introduces inherent limitations in scope and generalizability. The resulting knowledge graph is highly specialized but suffers from data homogeneity, as it primarily reflects findings from academic literature rather than integrating complementary real-world evidence. For instance, while publication data may highlight molecular interactions, they often lack essential clinical context (e.g., patient demographics, treatment outcomes, or adverse drug reactions). Additionally, this limited data representation may constrain the utility of AdDKG in systems biology or precision medicine applications. Furthermore, this reliance on curated literature introduces inherent biases, including a predominant coverage of English-language publications and the underreporting of negative results, which may skew the represented knowledge. It should also be noted that our approach, limited to titles and abstracts, is effective for identifying key findings but may miss the finer-grained biological insights and contextual depth provided by full-text analysis.

To overcome these limitations and enhance translational potential, four research directions should be prioritized.

i. Advanced relation extraction through hybrid architectures combining domain-specific language models (e.g., BioBERT [47]) with graph attention networks, specifically optimized to capture complex biomedical interactions (e.g., drug-protein-disease triplets).
ii. Multimodal knowledge integration employing federated learning frameworks to harmonize AdDKG with diverse data sources such as Electronic Health Records (EHRs), single-cell omics, clinical trial evidence, etc.
iii. Focused full-text mining through deep semantic models trained on specialized corpora to extract high-precision knowledge while minimizing noise and scalability issues.
iv. Dynamic knowledge maintenance implementing blockchain-based version control to enable traceable updates and ensemble validation against both structured databases (e.g., ClinVar [48]) and expert-curated benchmarks.

The successful implementation of these strategies would fundamentally shift AdDKG’s role. It would no longer be a static repository but an evolving, evidence-rich engine for translational discovery. This enables direct clinical applications, such as pinpointing candidates for drug repurposing by cross-referencing molecular mechanisms with clinical trial results, or identifying patient subpopulations most likely to respond to a specific therapy through the integration of EHRs and omics profiles, Together, these advancements pave the way for precision medicine in Alzheimer’s disease.

Looking ahead, the construction of AdDKG and its future enhancements point toward its integration into a broader ecosystem of complementary research tools. AdDKG is a literature-derived, semantically annotated knowledge base, making it a domain-specific resource for exploring targeted research questions. This core identity as a curated resource distinguishes it from general-purpose analytical tools and platforms. It is therefore positioned differently from macro-analytic bibliometric tools like CiteSpace, which excels at mapping large-scale research trends and landscapes [49]. Similarly, it differs from the general-purpose graph database platform like Neo4j, which offers flexibility for custom knowledge representation [50]. Instead, AdDKG provides a domain-specific, pre-integrated and query-ready knowledge resource generated through an automated pipeline. This distinction, far from being a limitation, establishes the foundation for their future synergy. A forward-looking research process could leverage CiteSpace to identify emerging frontiers, employ CI-er to build focused resources like AdDKG for deep semantic mining within those areas, and then utilize Neo4j to extend and share the resulting knowledge models. This integrated pipeline leverages and amplifies the unique strengths of each component to advance research.

## Conclusions

In this study, we developed a novel knowledge graph for AD and dementia (AdDKG) through semantic integration and annotation of relevant scientific literature. Using AdDKG’s semantic mining capabilities, we systematically investigated the interplay between astrocyte autophagy and pyroptosis in AD pathogenesis. Our analysis of 12 high-confidence publications revealed that genetic and environmental factors disrupt normal autophagic processes in astrocytes. This impairment leads to the pathological accumulation of both A$\beta$ and IL-1$\beta$, establishing a critical connection between astrocyte autophagy and pyroptosis in AD development. The resulting A$\beta$ accumulation initiates a destructive cascade: (*i*) compromising astrocytes’ ability to provide metabolic support to neurons, and (*ii*) triggering pyroptotic cell death in astrocytes. These events subsequently promote neuroinflammatory responses, p-Tau pathological, and ultimately neuronal death. These findings provide a mechanistic framework that highlights potential therapeutic targets for developing novel AD interventions.

## Supplementary Information

Supplementary Material 1.

Supplementary Material 2.

## Data Availability

The datasets used and analyzed during this study are available from the corresponding author upon reasonable request.
