# Supplementary material for: Astrocyte autophagy-pyroptosis crosstalk in Alzheimer’s: insights from knowledge graphs
Source: BMC Neurol. 2026 Mar 28;26:305. doi: 10.1186/s12883-026-04793-w (PMC13151352; doi:10.1186/s12883-026-04793-w)
Supplement: Supplementary file 2 — Supplementary Material 2. [file 12883_2026_4793_MOESM2_ESM.pdf]

Supplementary File

Astrocyte Autophagy-Pyroptosis Crosstalk in Alzheimer's: Insights from Knowledge Graphs

Ting Liu[1], Zhisheng Huang[2], Hongyun Qin[2*]

[1] Department of Central Laboratory, Gongli Hospital of Shanghai Pudong New Area, Miaopu Road, Shanghai, 200135, Shanghia, China.

[2*] Clinical Research Center for Mental Disorders, Shanghai Pudong New Area Mental Health Center, Tongji University School of Medicine, Sanlin Road, Shanghai, 200124, Shanghai, China.

[*]Corresponding author(s). E-mail(s): qinhongyun07@163.com;

Contributing authors: ting.liu.cn@hotmail.com; huang.zhisheng.nl@gmail.com;

Appendix A. Supplementary query examples for AdDKG

Example 1: Counting total publications

To quantify the total number of unique publications within AdDKG, we executed the query in SI Listing 1. The result, detailed in SI Figure 1, confirms that the database contains a total of 172,283 publications (identified by unique PubMed IDs and PMIDs).

SI Listing 1. Query protocol to count articles in AdDKG.

```
prefix rdf:<http://www.w3.org/1999/02/22-rdf-syntax-ns#>
prefix rdfs:<http://www.w3.org/2000/01/rdf-schema#>
prefix snomed:<http://www.ihtsdo.org/SCT_>
prefix addkg:<http://www.ztonebv.nl/KG\#>

SELECT DISTINCT (COUNT(distinct ?pmid) AS ?count)
WHERE {
        ?pmid    addkg:hasAnnotations    ?text.
        ?text    addkg:hasSource    "Title".
}
```

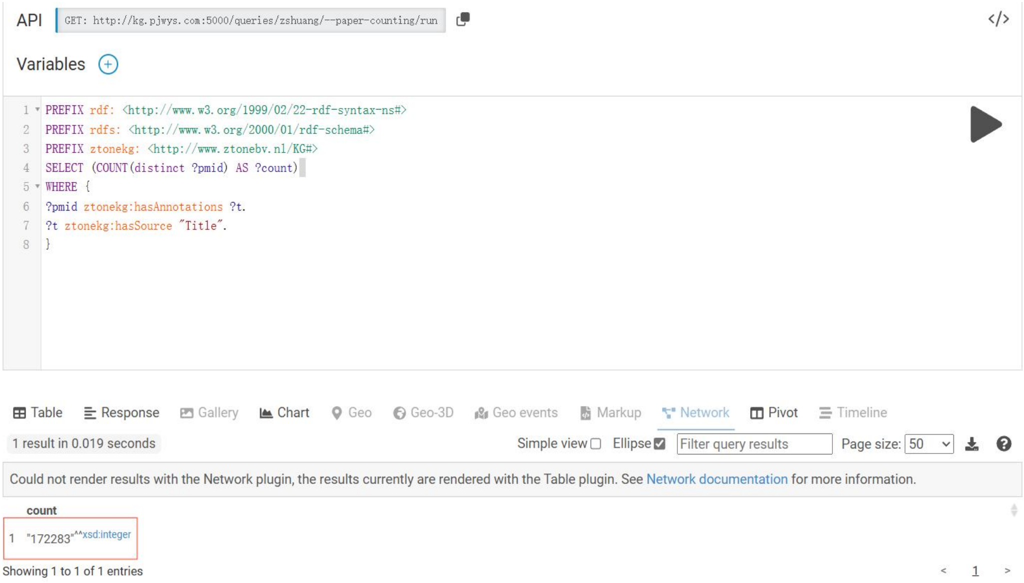

SI Figure 1. Numbers of articles collected in AdDKG.

Example 2: Tallying semantic concepts

To determine the number of concepts annotated in AdDKG, we designed the query code in Listing 2, by which the returned result revealed that the database contains a total of 148,864 concepts (as shown in SI Figure 2).

SI Listing 2. Query protocol to count concepts in AdDKG.

```
prefix rdf:<http://www.w3.org/1999/02/22-rdf-syntax-ns#>
prefix rdfs:<http://www.w3.org/2000/01/rdf-schema#>
prefix snomed:<http://www.ihtsdo.org/SCT_>
prefix addkg:<http://www.ztonebv.nl/KG\#>
prefix pubmed:<http://www.ncbi.nlm.nih.gov/pubmed/>

SELECT DISTINCT (COUNT(distinct ?conceptid) AS ?count)
WHERE {
      ?t1s1   addkg:SenseURL   ?conceptid.
}
```

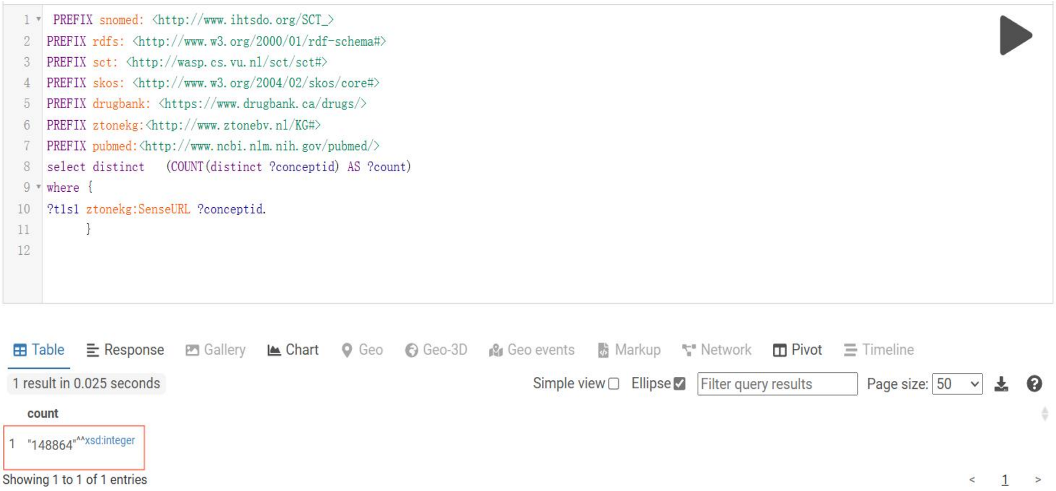

SI Figure 2. Numbers of concepts in AdDKG.

Example 3: Counting the frequency of concepts

We applied the query code shown in SI Listing 3 to calculate the frequency of the top 100 concepts in AdDKG. The results are presented in SI Figure 6, which displays the top 5 concepts.

SI Listing 3. Query protocol to count the frequency of the top 100 concepts in AdDKG.

```
prefix rdf:<http://www.w3.org/1999/02/22-rdf-syntax-ns#>
prefix rdfs:<http://www.w3.org/2000/01/rdf-schema#>
prefix snomed:<http://www.ihtsdo.org/SCT_>
prefix addkg:<http://www.ztonebv.nl/KG\#>
prefix pubmed:<http://www.ncbi.nlm.nih.gov/pubmed/>
prefix sct:<http://wasp.cs.vu.nl/sct/sct#>

SELECT DISTINCT (COUNT(distinct ?pubmed) AS ?count) ?conceptid ?concept
WHERE {
      ?t1s1   addkg:SenseURL   ?conceptid.
      ?conceptid   sct:hasEnglishPreferredLabel   ?concept.
      ?t1   addkg:hasSense   ?t1s1.
      ?s7   addkg:hasSenses   ?t1.
      ?s   addkg:hasTerm   ?s7.
      ?s1   addkg:hasAnnotation   ?s.
      ?pubmed   addkg:hasAnnotations   ?s1.
}
```

```
GROUP BY    ?conceptid    ?concept
ORDER BY DESC (?count)
LIMIT 100
```

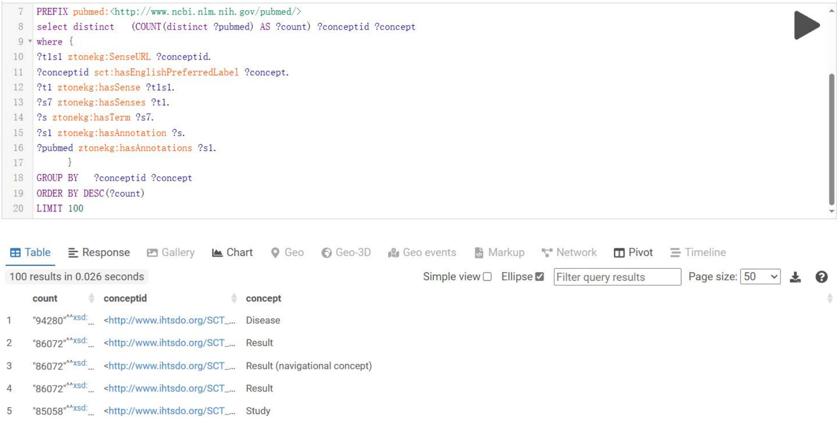

SI Figure 3. The top 100 concepts frequently annotated in AdDKG.

Example 4: Investigating dopamine in Alzheimer's disease

Given the role of neurotransmitter alterations in Alzheimer's disease, we identified articles concerning dopamine in AD. Using the SNOMED CT Concept ID for dopamine (35069000) in the SPARQL query shown in SI Listing 4, we retrieved 169 relevant papers (see SI Figure 4). The top 20 retrieved articles concerning dopamine in AD is presented in SI Figure 5.

SI Listing 4. Query protocol to retrieve reviews on dopamine in AdDKG.

```
prefix rdf:<http://www.w3.org/1999/02/22-rdf-syntax-ns#>
prefix rdfs:<http://www.w3.org/2000/01/rdf-schema#>
prefix snomed:<http://www.ihtsdo.org/SCT_>
prefix addkg:<http://www.ztonebv.nl/KG\#>
prefix pubmed:<http://www.ncbi.nlm.nih.gov/pubmed/>
prefix sct:<http://wasp.cs.vu.nl/sct/sct#>

SELECT DISTINCT ?pubmed ?title ?abstract
WHERE {
    ?conceptid   rdfs:subClassOf   snomed:35069000.
    ?t1s1   addkg:SenseURL   ?conceptid.
    ?conceptid sct:hasEnglishLabel   ?concept.
    ?t1   addkg:hasSense   ?t1s1.
    ?s7   addkg:hasSenses   ?t1.
    ?s   addkg:hasTerm   ?s7.
    ?s1   addkg:hasAnnotation   ?s.
    ?s1   addkg:hasSource   "Abstract".
    ?s1   addkg:hasText   ?abstract.
    ?pubmed   addkg:hasAnnotations   ?s1.
    ?pubmed   addkg:hasAnnotations   ?s1b.
    ?s1b   addkg:hasSource   "Title".
    ?s1b   addkg:hasText   ?title.
    FILTER regex (?title, "review","i")
}
```

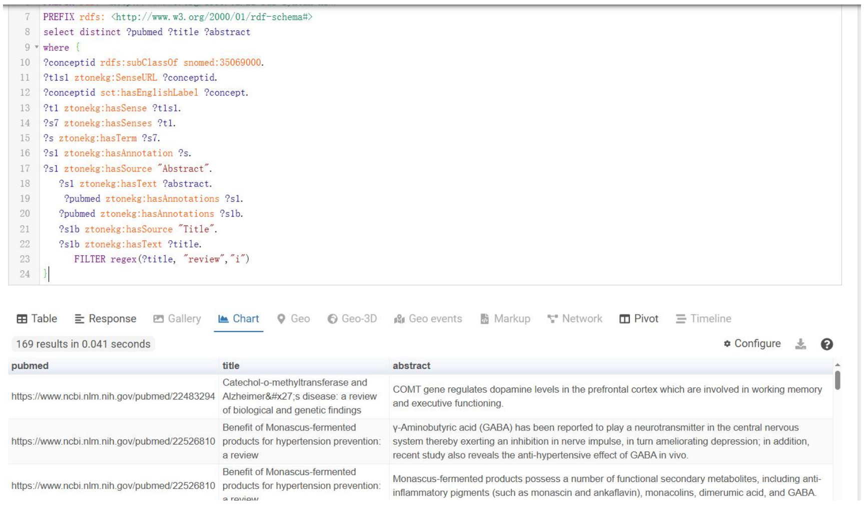

```
PREFIX rdfs: <http://www.w3.org/2000/01/rdf-schema#>
select distinct ?pubmed ?title ?abstract
where {
?conceptid rdfs:subClassOf snomed:35069000.
?t1s1 ztonekg:SenseURL ?conceptid.
?conceptid sct:hasEnglishLabel ?concept.
?t1 ztonekg:hasSense ?t1s1.
?s7 ztonekg:hasSenses ?t1.
?s ztonekg:hasTerm ?s7.
?s1 ztonekg:hasAnnotation ?s.
?s1 ztonekg:hasSource "Abstract".
   ?s1 ztonekg:hasText ?abstract.
    ?pubmed ztonekg:hasAnnotations ?s1.
   ?pubmed ztonekg:hasAnnotations ?s1b.
   ?s1b ztonekg:hasSource "Title".
   ?s1b ztonekg:hasText ?title.
      FILTER regex(?title, "review","i")
}
```


pubmed	title	abstract
https://www.ncbi.nlm.nih.gov/pubmed/22483294	Catechol-o-methyltransferase and Alzheimer's disease: a review of biological and genetic findings	COMT gene regulates dopamine levels in the prefrontal cortex which are involved in working memory and executive functioning.
https://www.ncbi.nlm.nih.gov/pubmed/22526810	Benefit of Monascus-fermented products for hypertension prevention: a review	γ-Aminobutyric acid (GABA) has been reported to play a neurotransmitter in the central nervous system thereby exerting an inhibition in nerve impulse, in turn ameliorating depression; in addition, recent study also reveals the anti-hypertensive effect of GABA in vivo.
https://www.ncbi.nlm.nih.gov/pubmed/22526810	Benefit of Monascus-fermented products for hypertension prevention: a review	Monascus-fermented products possess a number of functional secondary metabolites, including anti-inflammatory pigments (such as monascin and ankaflavin), monacolins, dimerumic acid, and GABA.

SI Figure 4. Retrieved articles concerning dopamine in AD retrieved from AdDKG..

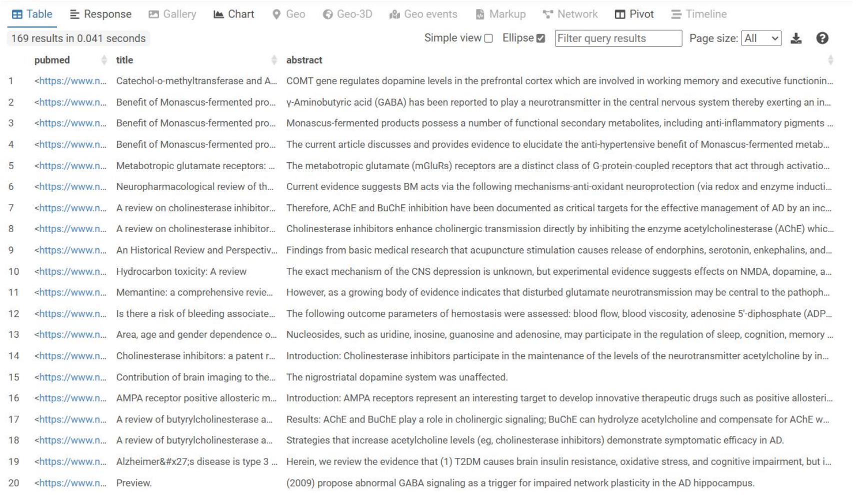

	pubmed	title	abstract
1	<https://www.n...	Catechol-o-methyltransferase and A...	COMT gene regulates dopamine levels in the prefrontal cortex which are involved in working memory and executive functionin...
2	<https://www.n...	Benefit of Monascus-fermented pro...	γ-Aminobutyric acid (GABA) has been reported to play a neurotransmitter in the central nervous system thereby exerting an in...
3	<https://www.n...	Benefit of Monascus-fermented pro...	Monascus-fermented products possess a number of functional secondary metabolites, including anti-inflammatory pigments ...
4	<https://www.n...	Benefit of Monascus-fermented pro...	The current article discusses and provides evidence to elucidate the anti-hypertensive benefit of Monascus-fermented metab...
5	<https://www.n...	Metabotropic glutamate receptors: ...	The metabotropic glutamate (mGluRs) receptors are a distinct class of G-protein-coupled receptors that act through activatio...
6	<https://www.n...	Neuropharmacological review of th...	Current evidence suggests BM acts via the following mechanisms-anti-oxidant neuroprotection (via redox and enzyme inducti...
7	<https://www.n...	A review on cholinesterase inhibitor...	Therefore, AChE and BuChE inhibition have been documented as critical targets for the effective management of AD by an inc...
8	<https://www.n...	A review on cholinesterase inhibitor...	Cholinesterase inhibitors enhance cholinergic transmission directly by inhibiting the enzyme acetylcholinesterase (AChE) whic...
9	<https://www.n...	An Historical Review and Perspectiv...	Findings from basic medical research that acupuncture stimulation causes release of endorphins, serotonin, enkephalins, and...
10	<https://www.n...	Hydrocarbon toxicity: A review	The exact mechanism of the CNS depression is unknown, but experimental evidence suggests effects on NMDA, dopamine, a...
11	<https://www.n...	Memantine: a comprehensive revie...	However, as a growing body of evidence indicates that disturbed glutamate neurotransmission may be central to the pathoph...
12	<https://www.n...	Is there a risk of bleeding associate...	The following outcome parameters of hemostasis were assessed: blood flow, blood viscosity, adenosine 5'-diphosphate (ADP...
13	<https://www.n...	Area, age and gender dependence o...	Nucleosides, such as uridine, inosine, guanosine and adenosine, may participate in the regulation of sleep, cognition, memory ...
14	<https://www.n...	Cholinesterase inhibitors: a patent r...	Introduction: Cholinesterase inhibitors participate in the maintenance of the levels of the neurotransmitter acetylcholine by in...
15	<https://www.n...	Contribution of brain imaging to the...	The nigrostriatal dopamine system was unaffected.
16	<https://www.n...	AMPA receptor positive allosteric m...	Introduction: AMPA receptors represent an interesting target to develop innovative therapeutic drugs such as positive allosteri...
17	<https://www.n...	A review of butyrylcholinesterase a...	Results: AChE and BuChE play a role in cholinergic signaling; BuChE can hydrolyze acetylcholine and compensate for AChE w...
18	<https://www.n...	A review of butyrylcholinesterase a...	Strategies that increase acetylcholine levels (eg, cholinesterase inhibitors) demonstrate symptomatic efficacy in AD.
19	<https://www.n...	Alzheimer's disease is type 3 ...	Herein, we review the evidence that (1) T2DM causes brain insulin resistance, oxidative stress, and cognitive impairment, but i...
20	<https://www.n...	Preview.	(2009) propose abnormal GABA signaling as a trigger for impaired network plasticity in the AD hippocampus.

SI Figure 5. The top 20 articles concerning dopamine in AD retrieved from AdDKG.

Example 5: Exploring the role of type 2 diabetes in AD

We used the SPARQL query in SI Listing 5 to investigate the articles regarding the role of type 2 diabetes and autophagy in AD. The query protocol returned 697 papers, as shown in SI Figure 6, in which the top 10 retrieved articles is presented.

SI Listing 5. Query protocol to retrieve articles relating type 2 diabetes and AD.

```
prefix rdf:<http://www.w3.org/1999/02/22-rdf-syntax-ns#>
prefix rdfs:<http://www.w3.org/2000/01/rdf-schema#>
prefix snomed:<http://www.ihtsdo.org/SCT_>
prefix addkg:<http://www.ztonebv.nl/KG\#>
prefix pubmed:<http://www.ncbi.nlm.nih.gov/pubmed/>
prefix sct:<http://wasp.cs.vu.nl/sct/sct#>

SELECT DISTINCT ?pubmed ?title ?abstract
WHERE {
        ?pubmed    addkg:hasAnnotations    ?s1.
        ?s1    addkg:hasSource    "Title".
        ?s1    addkg:hasText    ?title.
        FILTER REGEX (?title,"type 2 diabetes","i")
        ?pubmed    addkg:hasAnnotations    ?s1b.
        ?s1b    addkg:hasSource    "Abstract".
```

```
        ?s1b    addkg:hasText    ?abstract.
        FILTER REGEX (?abstract, "Autophagy", "i")
}
```

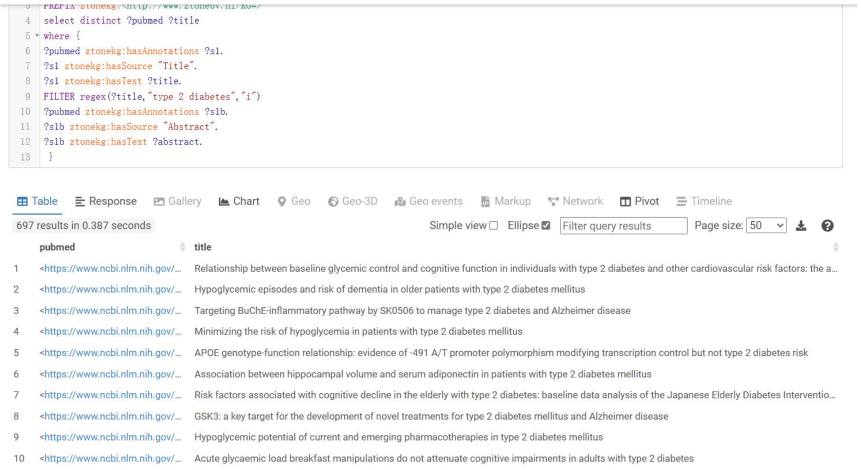

SI Figure 6. The returned results of the query protocol in SI Listing 5.
